# Once- Versus Twice-Daily Angiotensin-Converting Enzyme Inhibitors for Blood Pressure Control in Adult Patients With Hypertension

**DOI:** 10.7759/cureus.17331

**Published:** 2021-08-20

**Authors:** Kyle Fischer, Sandy Diec

**Affiliations:** 1 Pharmacy, University of Texas Southwestern Medical Center, Dallas, USA; 2 Pharmacy, University of Houston, College of Pharmacy, Houston, USA

**Keywords:** once- versus twice-daily dosing, ace inhibitors, hypertension, ambulatory blood pressure, blood pressure response

## Abstract

This review aimed to assess the efficacy and safety of once- versus twice-daily administration of angiotensin-converting enzyme (ACE) inhibitors for the management of hypertension. A literature search on PubMed and Google Scholar was performed (January 1980 to June 2020) using the following search terms: ACE inhibitors, lisinopril, enalapril, fosinopril, trandolapril, ramipril, perindopril, captopril, benazepril, ambulatory blood pressure, hypertension, twice-daily dosing, once-daily dosing. Reference lists from retrieved articles were examined for additional reports. Relevant English-language studies or those conducted in humans were considered. Overall, six studies were included that compared the efficacy of once-daily to twice-daily dosing of ACE inhibitors. Similar blood pressure-lowering effects, and, in some studies, greater blood pressure lowering has been noted in the twice-daily administration arm than once-daily administration of ACE inhibitors. ACE inhibitors' pharmacokinetic and pharmacodynamic properties play an integral role in determining the expected blood pressure-lowering outcome. It is noteworthy that adherence issues may arise when transitioning from a once-daily regimen to a twice-daily regimen. There appear to be no added safety concerns between twice-daily and once-daily administration of ACE inhibitors regarding safety outcomes. After reviewing the available literature, twice-daily dosing of ACE inhibitors may promote added blood pressure-lowering effects, with the advantages of reducing cost, reducing the risk of drug-drug interactions, reducing polypharmacy, and reducing patient confusion about their medications. Recommendations for twice-daily administration of ACE -inhibitors should be made via shared decision-making with the patient, and clinician judgment, to drive treatment selection.

## Introduction and background

Hypertension remains a significant public health challenge in the United States due to the substantial increase in cardiovascular disease (CVD) [1]. As one of the major modifiable risk factors for CVD, optimizing blood pressure control is critical in reducing heart disease and, ultimately, healthcare costs [2]. Many individuals newly diagnosed with hypertension are often started on a single antihypertensive agent and titrated to the maximum effective dose. The initiation of a new antihypertensive agent for elevated blood pressure depends on numerous factors such as age, concurrent medications, drug interactions, and comorbid conditions [3]. The 2017 American College of Cardiology (ACC)/American Heart Association (AHA) hypertension guidelines recommend four classes of agents as first-line therapy for managing essential hypertension, and among them are angiotensin-converting enzyme (ACE) inhibitors [3].

ACE inhibitors are one of the most commonly prescribed antihypertensive agents. ACE inhibitors exert their pharmacological effect by interfering with the renin-angiotensin-aldosterone system (RAAS) [4]. ACE inhibitors block the ACE enzyme, which prevents the conversion of angiotensin I to angiotensin II. With less angiotensin II circulating in the system, the effects observed are decreased blood pressure, increased natriuresis, and the prevention of smooth muscle and cardiac myocytes remodeling [4]. The antihypertensive efficacy is generalized throughout the ACE inhibitor class. Still, there are some differences regarding the pharmacodynamic and pharmacokinetic properties of the various ACE inhibitor agents. The pharmacokinetics of ACE inhibitors are essential in determining the plasma concentration profile and the agent's duration of effect [5]. Understanding ACE inhibitor pharmacokinetic data is vital to optimizing the dose and dose frequency best. ACE inhibitors generally exhibit a flat dose-response curve except for lisinopril, which indicates a linear dose-response curve [6]. ACE inhibitor doses commonly seen in the clinical setting are on the upper linear plateau of the concentration-effect curve. By titrating the dose, it is possible to prolong the apparent drug effect [5,6]. Fosinopril, ramipril, and trandolapril have been shown to have trough-to-peak effect ratios greater than 50% [6]. Although ACE inhibitors are typically dosed once daily, this suggests some ACE inhibitors' optimal frequency may be twice-daily administration [5-7].

In addition, previous landmark clinical trials demonstrated the efficacy and safety of primarily once-daily dosing of ACE inhibitors to treat hypertension [8-11]. However, these agents' pharmacodynamics and pharmacokinetics suggest that once-daily administration may not lead to a 24-hour effect [5,6,12,13]. Recent evidence also suggests that better blood pressure-lowering outcomes can be achieved when ACE inhibitors are scheduled in the evening compared to the morning [14-17]. The significance of administration timing may be due to the circadian variation that identifies the RAAS and its activation during nocturnal sleep [17]. Despite these findings, the question remains whether increased daily administration of ACE inhibitors will result in more significant blood pressure-lowering effects. This review aims to provide a concise overview of the current literature to elucidate any difference in efficacy and safety of once-daily compared to twice-daily dosing of ACE inhibitors. 

## Review

A literature search was conducted using PubMed and Google Scholar from January 1980 to June 2020 using the following search terms: ACE inhibitors, lisinopril, enalapril, fosinopril, trandolapril, ramipril, perindopril, captopril, benazepril, ambulatory blood pressure, hypertension, twice-daily dosing, once-daily dosing. Only articles in English that evaluated the efficacy of once-daily to twice-daily administration of ACE inhibitors were included. Reference lists from retrieved articles were examined for additional reports that evaluated once- versus twice-daily administration of ACE inhibitors for hypertension in adults. The authors selected the articles relevant to this review. A total of six studies were identified from the literature search that compared the efficacy of once-daily to twice-daily dosing of ACE inhibitors, as seen in Table 1. All studies were evaluated for scientific validity. Current guidelines do not provide a recommendation for the preferred timing of administration. Thus, we sought to explore whether or not twice-daily administration of ACE inhibitors is more efficacious than once-daily administration.

**Table 1:** Summary of Once-Daily Versus Twice-Daily Studies BP: blood pressure

Author (Year)	Trial Design	Participants (n)	Outcomes
Whelton et al. (1990) [18]	Multicenter, double-blind, parallel-group study	70	Efficacy: mean BP lowering for lisinopril once-daily: -10.7/-9.86 mm Hg. Mean BP lowering for captopril twice daily: -2.62/-5.95 mm Hg. Significant BP reductions in both systolic (p=0.023) and diastolic (p=0.007) measurements. Safety: no significant adverse reactions.
Poirier et al. (1993) [19]	Randomized double-blind, crossover study	34	Efficacy: trandolapril 0.5 mg twice-daily BP reduction: -7.88/-5.32 mm Hg (p=0.0001). Trandolapril 1 mg once-daily BP reduction: -5.84/-5.15 mm Hg (p=0.0018). Trandolapril 0.5 mg twice-daily showed a significantly (p=0.03) greater antihypertensive effect during sleep compared to trandolapril 1 mg once daily. Safety: no significant adverse reactions.
Girvin et al. (1999) [20]	Randomized single-blind, crossover study	25	Efficacy: enalapril 20 mg once-daily BP reduction: -13.5/-10.7 mm Hg. Enalapril 10 mg twice-daily BP reduction: -18.8/-11.7 mm Hg (p=0.068 for systolic and p=0.086 for diastolic BP). Safety: no safety data reported.
Goyal et al. (2007) [21]	Randomized prospective crossover study	32	Efficacy: ramipril 5 mg twice-daily BP reduction: -6.8/-4 mmHg (p<0.001). Ramipril 10 mg once-daily BP reduction: -8.6/-5 mm Hg (p<0.001). No statistically significant difference when comparing the two regimens' BP reduction. Safety: no safety data reported.
Szauder et al. (2015) [22]	Randomized unblinded prospective study	82	Efficacy: perindopril 4 mg twice-daily BP reduction: -15/-6 mm Hg. Perindopril 8 mg once-daily BP reduction: -13/-6 mm Hg. Perindopril 4 mg twice-daily showed a significant difference in the diurnal index (p<0.05). Safety: no safety data reported.
Tsai et al. (2017) [23]	Retrospective cohort study	146	Efficacy: lisinopril 40 mg once-daily BP reduction: -5.7/-1.3 mm Hg. Lisinopril 20 mg twice-daily BP reduction: -17.5/-6.6 mm Hg (p=0.0159). Lisinopril 20 mg twice-daily was associated with higher systolic BP reduction (OR, 9.1; 95% CI, 2.6-31.8 {p=0.0006}). Safety: two patients in the once-daily cohort discontinued lisinopril and one patient in the twice-daily cohort discontinued lisinopril due to adverse effects.

Lisinopril once daily versus captopril twice daily

One of the first studies that looked into the efficacy of twice-daily versus once-daily dosing of ACE inhibitors was done in 1990 [18]. Whelton and colleagues compared once-daily doses of 10, 20, and 40 milligrams (mg) lisinopril to twice-daily doses of 25, 50, and 100 mg of captopril [18]. Assessment of blood pressure was done via office readings 24 hours after lisinopril and 12 hours after captopril, then again at the end of the study at the four-week mark. There were no significant differences between baseline treatment means for systolic or diastolic office blood pressures for lisinopril (148/100 mm Hg) and captopril (148/99 mm Hg). At the end of the study, mean blood pressure lowering for lisinopril was 138 (-10.7)/88 (-9.86) mm Hg and for captopril was 144 (-2.62)/91 (-5.95) mm Hg. The findings of the study alluded to more significant blood pressure reductions in both systolic (p=0.023) and diastolic (p=0.007) measurements after analyzing the area under the curve of ambulatory blood pressure reductions. The authors reported that there were no significant adverse reactions that led to the discontinuation of treatment.

Trandolapril

In 1993, Poirier and colleagues presented their findings comparing trandolapril once-daily to twice-daily administration. The two groups compared consisted of 0.5 mg trandolapril twice daily and 1 mg once daily. The mean 24-hour blood pressure at baseline for the two groups was 146/93 mm Hg. There was a -7.88/-5.32 mm Hg reduction (p=0.0001) in the trandolapril 0.5 mg twice-daily group and a reduction of -5.84/-5.15 mm Hg in the trandolapril 1 mg once-daily group (p=0.0018 and p=0.0001). Between the two groups, there was not a statistical difference in reaching the goal diastolic blood pressure of equal or less than 90 mm Hg (trandolapril 1 mg once daily {36%} compared to 0.5 mg twice daily {42%}). However, the twice-daily regimen exhibited a more sustained reduction in ambulatory systolic blood pressure during sleep hours. While this was statistically significant, the authors questioned the clinical significance, stating that most patients with mild hypertension will have normal blood pressure during sleep. For safety, trandolapril once and twice daily was not associated with significant alterations in the laboratory and electrocardiographic parameters [19].

Enalapril

In 1999, Girvin and colleagues assessed the efficacy of enalapril 20 mg once daily to enalapril 10 mg twice daily [20]. Twenty-five patients were enrolled in the 16-week long study. The mean baseline blood pressure was 152.6/97.1 mm Hg. The two groups' overall sitting blood pressure-lowering values were reported as 139.1/86.4 mm Hg for the 20 mg once-daily arm and 133.8/85.4 mm Hg for the 10 mg twice-daily arm. Enalapril 10 mg twice daily demonstrated more significant blood pressure reduction (p=0.068 for systolic and p=0.086 for diastolic blood pressure) compared to enalapril 20 mg once daily. Although a statistical difference was not reached, the authors concluded that enalapril should be prescribed 10 mg twice daily, and adequate measures should be taken to improve patient compliance. The authors believed that the small sample size and increased non-compliance issues in the twice-daily regimen led to the non-statistically significant finding. There was no safety data reported for this study.

Ramipril

A substudy in 2007 stemming from the Heart Outcomes Prevention Evaluation (HOPE) trial recruited 29 patients to assess either a twice-daily dose of 5 mg versus a once-daily dose of 10 mg of ramipril. This study concluded a significant difference in reducing systolic blood pressure from an average of 123.7 mm Hg at baseline to 116.9 mm Hg on the twice-daily dose and 115.1 mm Hg on the once-daily dose. At baseline, diastolic blood pressure was 73.3 mm Hg and reduced to 69.3 mm Hg on the twice-daily dose and 68.3 mm Hg on the once-daily dose (p<0.001). There was no statistically significant difference between the two regimens; thus, the authors concluded that both regimens caused a significant blood pressure profile reduction [21]. When assessing safety with ramipril, three patients discontinued treatment due to adverse events, two patients developed a dry cough, and one patient reported dizziness. No safety reports comparing once-daily to twice-daily administration were reported.

Perindopril

One of the more recent studies published by Szauder and colleagues in 2015 assessed the blood pressure-lowering effects of once-daily evening administration of perindopril and losartan to twice-daily administration of perindopril and losartan. The mean baseline blood pressure was reported to be 151/88 mm Hg. In the perindopril 8 mg once daily arm, the 24-hour mean blood pressure was lowered to 138/82 mm Hg. In the perindopril 4 mg twice daily arm, the 24-hour blood pressure was reduced to 136/82 mm Hg. The authors reported no significant difference in blood-pressure-lowering effects between the four groups regarding the main systolic and diastolic values. However, perindopril 4 mg twice-daily showed a significant difference in the diurnal index (p<0.05) [22]. The diurnal index was used to calculate the difference between daytime and nighttime blood pressure expressed in percent and often distinguished between dippers and non-dippers. The authors concluded that twice-daily administration of perindopril 4 mg compared to once-daily administration of perindopril 8 mg is more effective at eliminating the non-dipper phenomenon. There were no safety outcomes reported in this study. 

Lisinopril

In 2017, Tsai and colleagues performed a retrospective cohort study, comparing the efficacy and safety of lisinopril 20 mg twice daily to lisinopril 40 mg once daily. The mean baseline blood pressure for the once-daily cohort was 150.7/85.3 mm Hg and 148.5/85.6 mm Hg for the twice-daily cohort. The study results alluded to a mean adjusted systolic blood pressure reduction of 10.2 mm Hg greater in the twice-daily cohort compared to the once-daily cohort (p=0.0159). Mean adjusted diastolic blood pressure reduction was 4.3 mm Hg greater in the twice-daily cohort compared with the once-daily cohort (p=0.0675). The authors concluded that twice-daily lisinopril dosing was associated with higher systolic blood pressure reduction than the once-daily frequency (OR, 9.1; 95% CI, 2.6-31.8 {p=0.0006}) [23]. The safety outcomes of this study included changes in potassium and serum creatinine, presence of acute kidney injury, any reported adverse events, and any discontinuation due to adverse effects. Three symptomatic adverse events were reported, two in the once-daily cohort (angioedema {n=1} and cough {n=1}) and one in the twice-daily cohort (dizziness {n=1}). The authors reported that serum potassium's mean change was 0.18 mEq/L in the once-daily cohort and 0.10 mEq/L in the twice-daily cohort. The mean change in serum creatinine was 0.17 mg/dL in the once-daily cohort and -0.08 mg/dL in the twice-daily cohort. Two patients in the once-daily cohort discontinued lisinopril due to adverse effects compared to one patient in the twice-daily cohort. While the authors presented safety outcomes, there were no statistical tests performed between the groups.

Discussion

Based on the literature review conducted, twice-daily dosing may be as effective as once-daily dosing of ACE inhibitors. Compared to once-daily dosing, there is a potential that twice-daily dosing may have a more significant blood pressure-lowering effect in adult patients. The six studies included in this review reported no substantial adverse effects or safety differences between twice-daily and once-daily administration [18-23]. Furthermore, the evidence may suggest this to be a class effect given that twice-daily dosing with lisinopril, enalapril, trandolapril, perindopril, captopril, and ramipril all demonstrated greater or similar blood pressure-lowering effects than once-daily administration of these agents [18-23].

There is also emerging evidence suggesting that the administration of antihypertensives at nighttime can improve overall blood pressure control and reduce major cardiovascular events in certain patients [14-17]. Studies have associated an increased risk of cardiovascular events in patients whose blood pressure does not dip at night [14,15]. Additionally, sleep blood pressure has shown to be a more sensitive prognostic marker of cardiovascular disease [24-29]. The RAAS peak activity occurs during sleep, which suggests that administering ACE inhibitors twice daily may further reduce sleep blood pressure while maintaining therapeutic efficacy on awake blood pressure [17,30-32]. Table 2 reviews the pharmacodynamics and pharmacokinetics of commonly prescribed ACE inhibitors.

**Table 2:** Pharmacodynamic and Pharmacokinetics of Commonly Prescribed ACE Inhibitors

Medication	Usual Dosing for Hypertension	Frequency of Administration	Pharmacodynamics/Pharmacokinetics	Adverse Reactions
Lisinopril [33]	5-40 mg	Once-daily	Onset of action: 1 hour. Duration: 24 hours. Half-life elimination: 12 hours. Time to peak: adults: around 7 hours	>10%: hypotension (4-11%), dizziness (4-11%); 1% to 10%: flushing, vasculitis, hyperkalemia, rash, vertigo, tinnitus, increased serum creatinine, cough; <1%: angioedema
Ramipril [34]	2.5-20 mg	Once-daily or twice-daily	Onset of action: 1-2 hours. Duration: 24 hours. Half-life elimination: 13-17 hours. Time to peak, serum: 1 hour	>10%: hypotension (11%), cough (7-12%); 1% to 10%: angina pectoris, syncope, headache, fatigue, hyperkalemia, increased serum creatinine; <1%: angioedema, tinnitus
Enalapril [35]	5-40 mg	Once-daily or twice-daily	Onset of action: around 1 hour. Duration: 12-24 hours. Half-life elimination: 11 hours. Time to peak, serum oral: 0.5-1.5 hours	>10%: increased serum creatinine (< 20%); 1% to 10%: hypotension, chest pain, dizziness, fatigue, rash, cough, hyperkalemia; <1%: angioedema, tinnitus
Benazepril [36]	5-40 mg	Once-daily or twice-daily	Onset of action: 1-2 hours. Duration: 24 hours. Half-life elimination: 10-11 hours. Time to peak: 0.5-1 hour	1% to 10%: hypotension, dizziness, headache, drowsiness; <1%: angioedema, nausea, rash
Captopril [37]	12.5-150 mg	Twice-daily or thrice-daily	Onset of action: within 15 minutes. Duration: dose-related, may require several weeks of therapy before the full hypotensive effect. Half-life elimination: around 1.7 hours. Time to peak: within 1-2 hours	1% to 10%: hypotension, chest pain, rash, hyperkalemia, increased serum creatinine, cough; <1%: angioedema, angina pectoris, dizziness, fatigue
Trandolapril [38]	1-4 mg	Once-daily	Onset of action: 1-2 hours. Duration: 72 hours after a single dose. Half-life elimination: around 6 hours. Time to peak: around 1 hour	>10%: hypotension (< 12%), dizziness (1-23%), cough (2-35%); 1% to 10%: syncope, hyperkalemia, hypocalcemia, weakness, increased serum creatinine, cerebral vascular accident; <1%: angioedema, flushing, gout, rash

Although there is a paucity of data regarding the direct comparison of twice-daily to once-daily administration of ACE inhibitors, this review provides weak evidence supporting the efficacy and safety of twice-daily administration. At this time, switching an ACE inhibitor from once-daily to twice-daily frequency before adding on additional therapy may promote added blood pressure lowering effects. The 2017 ACC/AHA hypertension guidelines recognize that many patients may need more than one antihypertensive medication to control blood pressure [3]. However, switching to twice-daily dosing of an ACE inhibitor for patients near blood pressure goals can potentially delay the initiation of another antihypertensive agent. Additional benefits may include minimization in cost, drug-drug interaction risks, polypharmacy, and patient confusion. There are potential disadvantages to switching to twice-daily dosing of ACE inhibitors. Twice-daily administration may lead to adherence issues. Therefore, patients that have exhibited high adherence to twice-daily regimens, patients with uncontrolled blood pressure that do not want to add on another medication, or those with blood pressure that is difficult to control may be suitable candidates.

Some limitations exist with this review. A small sample size from most of the trials mentioned may limit generalizability. In addition, most of the trials were conducted in a single center, and one trial was a retrospective cohort design. A future randomized multicenter prospective trial that evaluates 24-hour ambulatory blood pressure monitoring and evaluating adherence could help overcome these limitations and aid in a stronger recommendation.

## Conclusions

The current treatment guidelines do not provide any recommendation for twice-daily administration over once-daily administration. However, several studies presented have demonstrated similar efficacy with no additional safety concerns. This review article demonstrates that in patients with hypertension, the use of twice-daily dosing of ACE-inhibitors may provide similar and in some cases further blood pressure reduction than once-daily dosing. Consideration to recommend twice-daily dosing of ACE-inhibitors should be based on shared decision-making with each patient and clinician's assessment on the indication, efficacy, safety, and convenience.
